# Reframing High School Dropout as a Public Health Issue [Response to Letters]

**Published:** 2008-03-15

**Authors:** Nicholas Freudenberg, Jessica Ruglis

**Affiliations:** Hunter College School of Health Sciences, City University of New York, New York, New York; Graduate Center, City University of New York, New York, New York

## In Reply:

We agree with Dr. Fiscella (1) that school readiness is an appropriate measure for educational success and health and that early childhood interventions are an important component of the portfolio of interventions needed to improve educational outcomes in the United States, which lags behind other developed nations in its commitment to early childhood education (2). Developing objective and meaningful measures of school readiness without replicating the obsession with testing that characterizes the "No Child Left Behind" mandates is a challenge (3).

We also agree with Dr. Appleton-Arnaud (4) that helping parents through adult education and English-as-second-language programs could improve child, family, and community health.

However, we emphasize that, in our view, no single type of intervention in a single setting or at a single developmental stage can by itself create opportunities for improving health by improving education, or vice versa. By linking a wide array of educational, health, and other interventions, we can promote both good health and educational achievement, and we can reduce the unconscionable socioeconomic and racial/ethnic disparities in both health and education.
